# Fresh or frozen day 6 blastocyst transfer: is there still a question?

**DOI:** 10.1186/s12958-024-01214-w

**Published:** 2024-04-24

**Authors:** Lucile Ferreux, Mathilde Bourdon, Ahmed Chargui, Julie Firmin, Chloé Maignien, Pietro Santulli, Catherine Patrat, Khaled Pocate-Cheriet

**Affiliations:** 1 Hôpitaux de Paris (AP- HP), APHP, Centre – Université de Paris Cité, Hôpital Cochin, Service de Biologie de la Reproduction-CECOS, Paris, France; 2 Hôpitaux de Paris (AP–HP), AP-HP, Centre–, Université de Paris Cité, Hôpital Cochin, Service de Gynécologie-Obstétrique II et de Médecine de la Reproduction, Paris, France; 3 Université de Paris Cité, Institut Cochin, U1016, INSERM, CNRS, Paris, F-75014 France

**Keywords:** Assisted reproductive technologies, Single frozen blastocyst transfer, Prolonged embryo culture, Day 6 blastocyst

## Abstract

**Background:**

The Live Birth Rate (LBR) after day 5 (D5) blastocyst transfer is significantly higher than that with D6 embryos in both fresh and frozen-vitrified embryo transfer cycles, according to the most recently published meta-analyses. Therefore, for women obtaining only D6 blastocysts, the chances of pregnancy may be lower but nonetheless sufficient to warrant transferring such embryos. The best strategy for transfer (i.e., in fresh versus frozen cycles) remains unclear and there is a paucity of data on this subject.

**Methods:**

A total of 896 couples with D6 single blastocyst transfers were retrospectively analyzed: patients receiving a fresh D6 embryo transfer (Fresh D6 transfer group, *n* = 109) versus those receiving a frozen-thawed D6 embryo transfer (Frozen D6 transfer group, *n* = 787). A subgroup comprising a freeze-all cycle without any previous fresh or frozen D5 embryo transfers (Elective frozen D6, *n* = 77) was considered and also compared with the Fresh D6 transfer group. We compared LBR between these two groups. Correlation between D6 blastocyst morphology according to Gardner’s classification and live birth occurrence was also evaluated. Statistical analysis was carried out using univariate and multivariate logistic regression models.

**Results:**

The LBR was significantly lower after a fresh D6 blastocyst transfer compared to the LBR with a frozen-thawed D6 blastocyst transfer [5.5% (6/109) vs. 12.5% (98/787), *p* = 0.034]. Comparison between LBR after Elective frozen D6 group to the Fresh D6 blastocyst transfers confirmed the superiority of frozen D6 blastocyst transfers. Statistical analysis of the blastocyst morphology parameters showed that both trophectoderm (TE) and inner cell mass (ICM) grades were significantly associated with the LBR after D6 embryo transfer (*p* < 0.001, *p* = 0.037). Multiple logistic regression revealed that frozen D6 thawed transfer was independently associated with a higher LBR compared with fresh D6 transfer (OR = 2.54; 95% CI: [1.05–6.17]; *p* = 0.038). Our results also show that transferring a good or top-quality D6 blastocyst increased the chances of a live birth by more than threefold.

**Conclusions:**

Our results indicate that transferring D6 blastocysts in frozen cycles improves the LBR, making it the best embryo transfer strategy for these slow-growing embryos.

**Clinical trial number:**

Not applicable.

## Introduction

The goal of in vitro fertilization (IVF) is to achieve the live birth of a healthy child in infertile patients. This implies the transfer of a selected good-quality embryo to a receptive endometrium. In recent years, prolonged embryo culture has enabled self-selection of the most competent embryos. The advent of vitrification for cryopreservation has enhanced the success of embryo transfer at the blastocyst stage, thereby promoting the single-embryo transfer policy [1]. A recent meta-analysis showed that the transfer of blastocysts developing on day 5 (D5) is associated with a significantly higher live birth rate (LBR) than with day 6 (D6) blastocysts in both fresh and frozen cycles [2]. Moreover, when fresh versus frozen cycles were analyzed together or separately, the transfer of slow-growing D6 embryos was associated with a higher risk of miscarriage compared with that of D5 embryos [2]. These findings could be partially related to higher aneuploidy rates observed in D6 blastocysts [3, 4]. However, even comparison of similarly classified (Gardner classification) D5 and D6 frozen euploid blastocyst transfers showed that the D6 blastocyst group had a lower implantation potential [5], thus suggesting that processes other than endometrium synchronicity and aneuploidy may also contribute to lower clinical outcomes of transfers of slower D6 blastocysts.

In clinical practice, for patients obtaining both D5 and D6 blastocysts, transferring D5 blastocysts first is the most reliable strategy to achieve pregnancy and live birth. For patients for whom only slower D6 blastocysts have been obtained, these embryos should still be considered for transfer because they can lead to pregnancy and live birth despite their lower implantation potential. In this context, the question of the D6 blastocyst transfer with a fresh or frozen cycle remains a matter of debate. Indeed, according to some authors, transferring such embryos in frozen cycles leads to higher clinical outcomes compared with those obtained in fresh cycles [6] while for others, transferring D6 blastocysts in fresh cycles remains the best option [7].

In this context of controversy, the aim of this study was to compare live birth rates between fresh versus frozen D6 embryo transfers, taking into account confounding factors, in an effort to identify the best embryo transfer strategy for improving live birth rates with slow-growing blastocysts. In addition, focusing on frozen D6 blastocysts, we sought to define whether morphological criteria, according to Gardner’s classification, can assist in the selection of the blastocysts to be transferred.

## Materials and methods

We conducted an observational, retrospective, cohort study that included infertile patients for whom a blastocyst transfer after an IVF/intracytoplasmic sperm injection (ICSI) cycle was performed in our Assisted Reproductive Technology (ART) center between 01/01/2018 and 01/07/2022. The inclusion criteria were: women ≤ 42 years of age, having one or more slow-growing blastocysts expanded on D6 available for transfer; and a single D6 blastocyst transfer (≥ B3 according to the Gardner classification). The exclusion criteria were as follows: embryo lysis after thawing, double embryo transfer, cycle canceled for personal reasons, or inappropriate endometrial thickness (< 6 mm). Patients were only included once in the analysis.

The study was approved by local ethics committees (Ethics Committee of Cochin Hospital, Research License AAA-2023-09021) and the National Data Protection Authority (Commission Nationale de l’Informatique et des Libertés, CNIL n°1,988,293 v0).

### Data collection

After a medical consultation, the following data were prospectively collected for the patients: age (years), IVF/ICSI rank, type of infertility (primary or secondary), infertility causes (e.g., ovulatory disorder, male factor, tubal factor, endometriosis, premature ovarian failure, or idiopathic) [8], body mass index (BMI), smoking habits, day 3 FSH and estradiol, antral follicle count (AFC), and AMH (Anti-Müllerian Hormone) level.

### Controlled ovarian stimulation (COS) protocol

Women were stimulated, monitored, and managed according to our institutional clinical protocols as reported previously [9]. Final oocyte maturation was triggered when ≥ 3 ovarian follicles with a diameter of ≥ 17 mm were visible by ultrasound and when the estradiol (E2) levels were ≥ 1000 pg/mL. Final oocyte maturation was achieved using either a single injection of 0.2 mg of gonadotropin-releasing hormone (GnRH) agonist (triptorelin, Decapeptyl®, Ibsen France) or 250 µg of recombinant human chorionic gonadotropin (rhCG) (Ovitrelle, Serono, France). Oocyte retrieval was performed under vaginal ultrasound guidance 36 ± 2 h later.

### Fertilization methods and embryo culture and scoring

After retrieval, oocytes were washed and incubated in 50 µL of IVF medium droplets for 2–3 h at 37 °C (IVF, CooperSurgical, USA). IVF or ICSI was performed according to biological and clinical indications. Cumulus–oocyte complex (COC) was conventionally inseminated with nearly 6,000 motile spermatozoa/COC. Before ICSI, cumulus cells were removed mechanically after exposure to hyaluronidase (80 IU/mL) (FujiFilm, Irvine Scientific, USA). Only metaphase II oocytes were injected. Fertilization was assessed 16–18 h post insemination/injection, and zygotes were individually maintained in extended culture until the blastocyst stage in 40 µL of CSCC medium droplets (FujiFilm, Irvine Scientific, USA) under mineral oil (FujiFilm, Irvine Scientific, USA) and incubated at 37 °C in a controlled atmosphere (5.5% CO_2_, 5% O_2_). At the blastocyst stage, embryo quality was assessed on D5 and D6 according to the Gardner classification, which takes into account the expansion grade as well as the inner cell mass (ICM) and trophectoderm (TE) development [10]. Based on this classification, only expanded or hatching top- (B3 AA, B4 AA, and B5 AA), good- (B3 AB/BA/BB, B4 AB/BA/BB, and B5 AB/BA/BB), and fair- (B3 AC/BC/CA/CB, B4 AC/BC/CA/CB, and B5 AC/BC/CA/CB) quality embryos were selected for transfer and cryopreservation by vitrification. Only expanded blastocysts (≥ B3) were transferred during the study period because of our embryo observation timing policy. Indeed, before embryo transfer or vitrification, blastocysts were observed at D5 in the morning as well as in the afternoon for those that were between grades B2 and B3 in the morning. Slowly developing blastocysts at D5 (B1 and B2 grade) were maintained in culture until D6 for fresh transfer, cryopreservation, or destruction.

### Blastocyst vitrification and thawing procedures

Blastocyst vitrification was performed using closed CBS-VIT High Security (HS) straws (Cryo Bio System, France) in combination with DMSO-EG-S as the cryoprotectants (Vitrification Freeze Kit, FujiFilm, Irvine Scientific, USA). The first step of the vitrification procedure consisted of exposing the embryos to cryoprotectants, and this was carried out at room temperature (approximately 20 °C). Each blastocyst was first incubated for 1 min in a 50 µL droplet of HEPES-buffered culture medium. It was then transferred into two 50 µL droplets of diluted equilibration solution (ES) containing 7.5% (v/v) DMSO and 7.5% (v/v) ethylene glycol, and incubated for 2 min in each droplet before being transferred into a third 50 µL droplet of ES solution and incubated for a further 10 min. The blastocyst was then transferred consecutively into four 25 µL droplets of vitrification solution (VS) containing 15% (v/v) DMSO, 15% (v/v) ethylene glycol, and 0.5 M sucrose and then immediately loaded onto the CBS-HS straw. Each straw containing a single blastocyst was then sealed and plunged into liquid nitrogen.

On the day of embryo transfer, the selected blastocyst with the highest score was thawed using an Irvine Scientific Thaw Kit (FujiFilm, Irvine Scientific© Thaw Kit, USA). A Petri dish containing one 300 µL droplet of thawing solution (TS: 1.0 M sucrose in HEPES-buffered HTF medium) was heated and maintained at 37 °C. The straw was transferred from the liquid nitrogen storage container. After cutting the straw and pulling out the capillary from the straw, the gutter was immediately placed in the heated TS droplet, allowing the blastocyst to be released from the gutter and maintained at 37 °C for 1 min. The blastocyst was then incubated twice for 1 min at room temperature in two 50 µL TS droplets and transferred to the first of two 50 µL dilution solution droplets (DS: 0.5 M sucrose in HEPES-buffered HTF medium) followed by a 2 min incubation in a second DS droplet. Finally, the blastocyst was washed in three droplets (50 µL each) of wash solution (HEPES-buffered HTF medium) for 3 min. The blastocyst was then transferred into a CSCC culture medium (CSCC, FujiFilm, Irvine Scientific, USA) droplet in a culture dish. After warming, the blastocyst quality was evaluated when re-expansion occurred, in which case the quality was always the same as before vitrification. In case of ICM and/or trophectoderm lysis, the blastocyst was discarded and another one was warmed when available. After survival assessment, the blastocyst was maintained in an incubator before intrauterine transfer 2–4 h hours later under ultrasound guidance.

### Endometrial preparation before embryo transfer (ET)

For fresh ET, the women began progesterone treatment (800 mg vaginal capsule, Utrogestan® Besins International, Montrouge, France), the day of the oocyte retrieval (i.e. fresh transfer occur on the 6th day of progesterone exposure), and E2 was delivered transdermally (2 mg/day, through two 100 systems simultaneously, Femsept® Theramex, La Defense, France) one day before the ET.

For frozen ET, the endometrium was prepared either with (i) hormonal replacement therapy (HRT) preceded by a degree of down-regulation with a GnRH agonist (a single dose of 11.25 mg IM, Decapeptyl®; Ipsen Pharma, Boulogne-Billancourt, France), or (ii) a modified natural or (iii) a stimulated cycle regimen. The protocol for HRT cycles has been described previously [11]. Briefly, progesterone supplementation began five days before the embryo transfer with vaginal progesterone at 800 mg daily (Utrogestan®, Besins International, Montrouge, France) without any change in the progesterone dose during the treatment. The last dose before the embryo transfer was administered on the morning of the embryo transfer (between 7 and 9 a.m.). The blastocysts were warmed on the day of transfer, i.e., on the 6th day of progesterone exposure. For a stimulated cycle, 25–75 IU/day of recombinant follicle-stimulating hormone (FSH) or urinary FSH (hMG) was used from day 4 of the cycle. For the modified natural and stimulated ovulatory cycle regimens, ovulation was determined by ultrasound monitoring as soon as the leading follicle was larger than 16 mm. Final oocyte maturation was achieved using rhCG triggering (Ovitrelle®; Merck Serono, Lyon, France). Vaginal progesterone at a dose of 800 mg per day (Utrogestan®, Besins International) was administrated for luteal phase support, starting 36 h after ovulation triggering. Women who became pregnant by these procedures continued with the same dose of progesterone (P) (and E2 treatment in HRT cycles) until 12 weeks of gestation.

### Data analysis and statistics

For the purpose of this study, two groups were compared: (i) a group of women who received a fresh D6 blastocyst transfer (Fresh D6 group), defined as the first fresh embryo transfer, and (ii) a group composed of women who underwent a frozen-thawed D6 blastocyst transfer (Frozen overall D6 group), defined as the first warming cycle of a D6 blastocyst with possible previous fresh or frozen D5 embryo transfer. Among these D6 first warming cycles, a subgroup called “Elective frozen D6” consisting of a freeze-all cycle without any previous fresh or frozen D5 embryo transfers was analyzed and also compared with the 109 fresh D6 transfers.

The primary outcome was the live birth rate after a single D6 blastocyst transfer. A live birth corresponded to the delivery of a viable infant at ≥ 28 weeks of gestation after embryo transfer [12]. The secondary outcomes included the clinical pregnancy (cPR) and early miscarriage rates. Clinical pregnancy was determined by ultrasonographic documentation of at least one fetus with a heartbeat at 6–7 weeks of gestation [13]. The early miscarriage rate (EMR) was defined as pregnancy loss occurring after confirmed cPR, as an intrauterine pregnancy ≤ 10 weeks based on size by ultrasound [14].

The data were entered into a digital database and analyzed using SPSS software (SPSS Inc., Chicago, IL, USA). A p-value < 0.05 was considered to be statistically significant. For the univariate statistical analysis, we used specific tests: Pearson’s χ2 or Fisher’s exact test for the qualitative variables and the Kruskal–Wallis or the Mann–Whitney test for the quantitative variables, as appropriate. A logistic regression analysis was performed to determine the variables that could be independently associated with the LBR and that could affect outcomes. The variables associated with a live birth occurrence at a threshold of *p* ≤ 0.10 in univariate analysis and those clinically relevant were retained for the multivariate analysis. Correlation between the baseline characteristic variables was tested, and, if two variables were highly correlated, only one of them was introduced in the model. The parameter values for each of the final models were determined by the maximum likelihood method. In case of significant differences, odds ratios (OR) and their 95% confidence intervals (CI 95%) were calculated from the model’s coefficients and their standard deviations.

## Results

### Study population

**Fig. 1:**
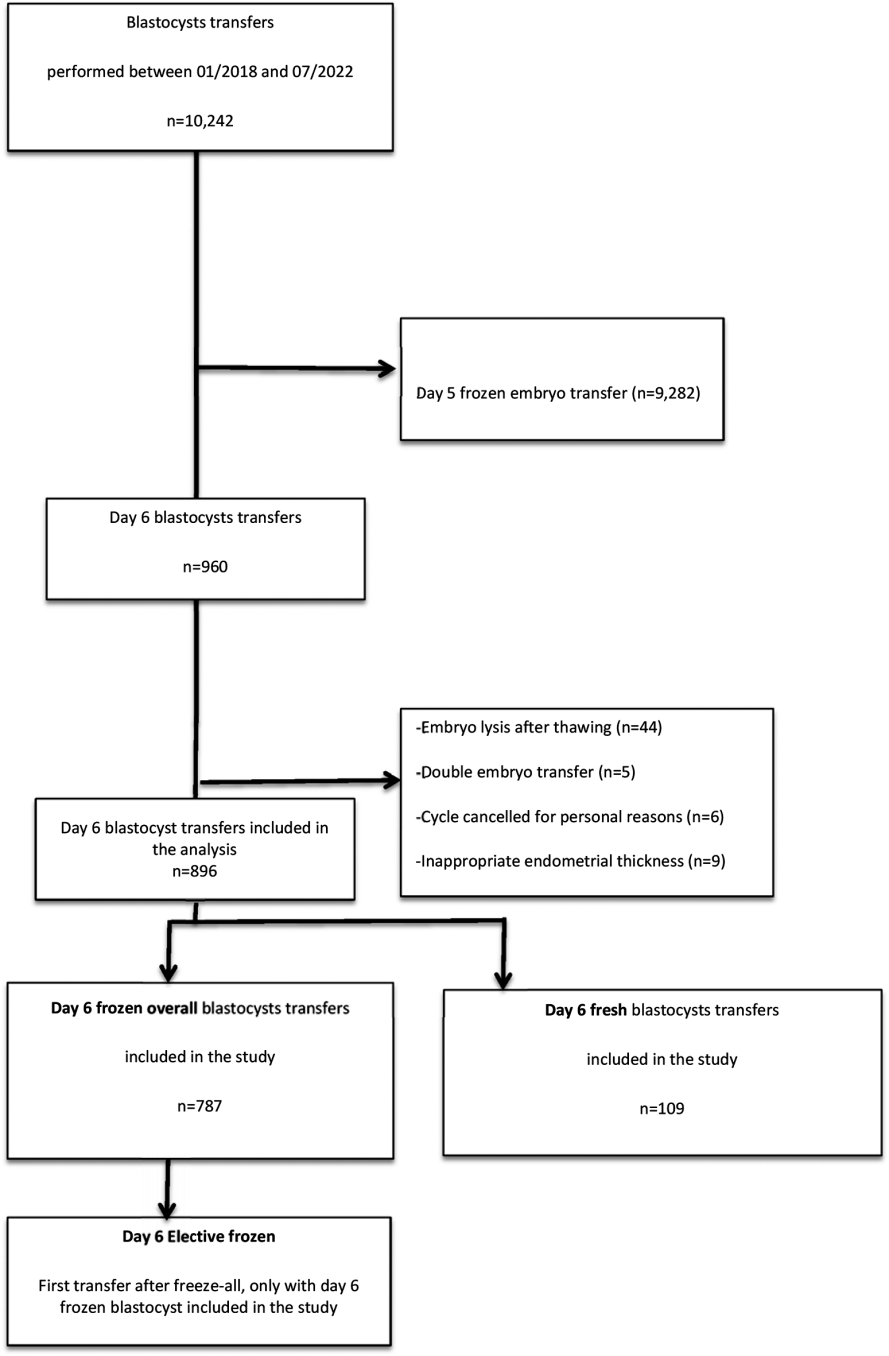
Fresh versus frozen-thawed day 6 blastocysts transfer cycles inclusion flowchart

In total, 896 single D6 blastocyst transfers were analyzed in this study. They were divided into 109 fresh D6 blastocyst transfers (Fresh D6) and 787 frozen-thawed D6 blastocyst transfers (Frozen overall D6) (Fig. 1). Among these D6 warming cycles, a subgroup of 77 first frozen-thawed D6 blastocyst transfers (called “Elective frozen D6”) was also compared to the 109 fresh D6 transfers.

The patient baseline characteristics for the two groups at the time of transfer are presented in Table 1. The women’s mean age, body mass index (BMI), and smoking habits did not differ between the two groups. There were no differences concerning the proportion of either top- or good-quality blastocysts transferred. The patient ovarian reserves were higher in the frozen D6 group compared with that of the fresh D6 group when considering the AMH level (2.8 ± 2.2 ng/mL versus 1.8 ± 1.4 ng/mL, respectively; *p* < 0.001) and AFC (17.1 ± 10.8 versus 13.9 ± 7.5, respectively; *p* = 0.006), and conversely, the endometrial thickness on the first day of progesterone administration was significantly lower in the frozen D6 group compared with that of the fresh D6 group (9.0 ± 1.9 mm versus 9.4 ± 2.6 mm, respectively; *p* = 0.035).

**Table 1:** General characteristics of the study population (*n* = 896)

	D6 frozen blastocyst transfers (*n* = 787)	D6 fresh blastocyst transfers (*n* = 109)	P-value
**Mean age at retrieval (y.o.)**	36.1 ± 4.1	35.9 ± 4.4	0.966 ^mw^
**Smoking habits (%)**	82 (10.4)	14 (12.8)	0.442^k^
**BMI (kg/m** ^**2**^ **)**	23.6	23.8	0.485 ^mw^
**Type of infertility**			0.123^k^
Primary	528 (67.1)	65 (59.6)	
Secondary	259 (32.9)	44 (40.4)	
Main causes of infertility*			
Endometriosis	346 (44.0)	38 (34.9)	0.072^k^
Male factor	299 (38.0)	48 (44.0)	0.225^k^
Tubal factor	145 (18.4)	12 (11.0)	0.056^k^
Ovulatory disorders	48 (6.1)	3 (2.8)	0.158^k^
Idiopathic	70 (8.9)	13 (11.9)	0.309^k^
**Ovarian reserve**			
Day 3 FSH (IU/L)	7.1 ± 2.3	7.6 ± 2.9	0.132 ^mw^
Day 3 estradiol (pg/mL)	51.1 ± 84.3	47.1 ± 19.2	0.349 ^mw^
AMH (ng/mL)	2.8 ± 2.2	1.8 ± 1.4	< 0.001 ^mw^
AFC (mean)	17.1 ± 10.8	13.9 ± 7.5	0.006 ^mw^
**IVF/ICSI rank (n)**	1.6 ± 1.1	1.9 ± 1.1	< 0.001 ^mw^
**Endometrial thickness (mm)**	9.0 ± 1.9	9.4 ± 2.6	0.035 ^mw^
**Top- or good-quality embryo transfer** ^a^	542 (68.9)	82 (75.2)	0.176^k^
**Top-quality embryo** ^a^	26 (3.3)	0 (0.0)	0.054^k^
**Type of endometrial preparation (FET cycle only) (%)**			*NA*
HRT cycle	547 (69.6)	*NA*	
HRT cycle + downregulation with GnRHa	152 (19.3)	*NA*	
Natural cycle	72 (9.1)	*NA*	
Stimulated cycle	16 (2.0)	*NA*	

D6 blastocyst = day 6 blastocyst; y.o., years old; IVF/ICSI, in vitro fertilization/intracytoplasmic sperm injection; AFC, antral follicle count; AMH, anti-Müllerian hormone; BMI, body mass index; FET, frozen embryo transfer; FSH, follicle-stimulating hormone; HRT, hormonal replacement therapy

*Several associated infertility causes for some patients

a – A top-quality embryo was defined as a B3–B4 or B5 embryo = AA, and a good-quality embryo was defined as a B3–B4 or B5 embryo ≥ BB (AB, BA, or BB) according to the grading scale proposed by Gardner; *Couples can have several causes of infertility

The data are presented as means ± the standard error or n (%) unless specified otherwise. mw Mann–Whitney t-test; k Pearson’s chi-square test

### ART outcomes

**Fig. 2:**
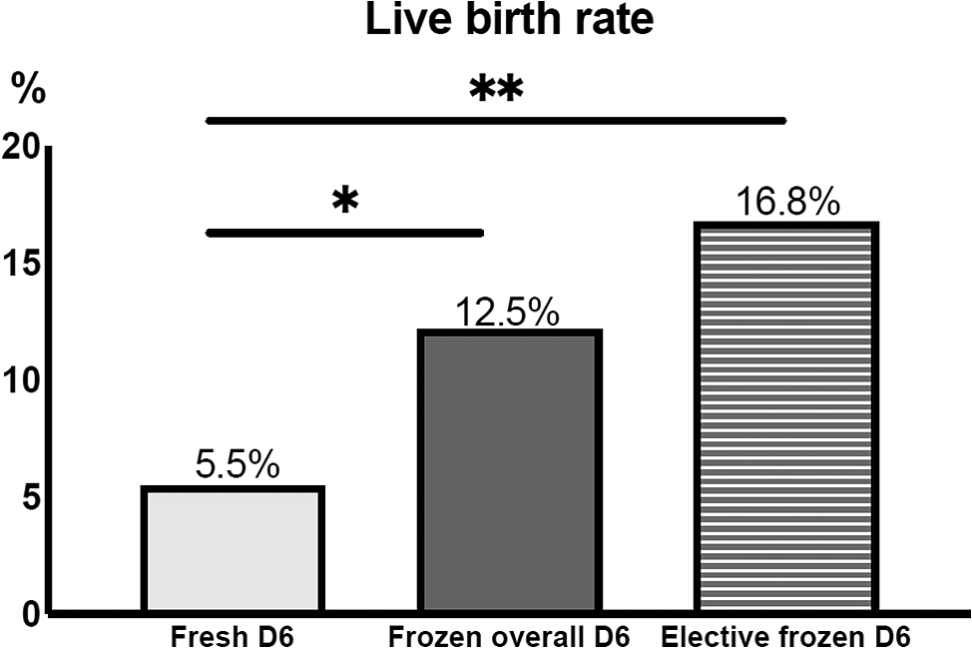
Comparison of live birth rate following fresh D6 versus frozen overall D6 or elective frozen D6 blastocysts transfer (*p < 0.05; **p < 0.001). Frozen overall D6 group = 1st warming cycle of a D6 blastocyst with possible previous fresh or frozen D5 embryo transfer. Elective frozen D6 = a subgroup among the Frozen overall D6 group that consisted of a 1st warming cycle of a D6 blastocyst, during a freeze-all cycle without any previous fresh or frozen D5 embryo transfers

The live birth rate (LBR) was significantly higher in the frozen overall D6 group compared with the fresh D6 group [98/787 (12.5%) vs. 6/109 (5.5%), respectively; *p* = 0.034] (Table 2) but also when considering elective D6, i.e., the 1st transfer compared with the fresh D6 transfer group [13/77 (16.8%) vs. 6/109 (5.5%), respectively; *p* < 0.001) ] (Fig. 2). The cPR was also higher following frozen overall D6 transfer compared with the fresh D6 transfer cycle [134/787 (17.0%) vs. 8/109 (7.3%), respectively; *p* = 0.009]. However, no significant difference was observed between the two groups regarding EMR [36/134 (26.9%) vs. 2/8 (25.0%), respectively; *p* = 0.908].

**Table 2:** Frozen versus fresh D6 embryo transfers outcomes

	Frozen D6 blastocyst transfers (*n* = 787)	Fresh D6 blastocyst transfer (*n* = 109)	p-value
**Clinical PR**	134 (17.0)	8 (7.3)	0.009
**Early miscarriage rate**	36 (26.9)	2 (25.0)	0.908
**Total live birth rate**	98 (12.5)	6 (5.5)	0.034

D6 = day 6; PR, pregnancy rate

The data are represented as means ± the standard error or n (%) unless specified otherwise

^k^ Pearson’s chi-square test

A multivariate analysis was performed to adjust for potentially confounding factors such as the women’s age at oocyte retrieval, fresh or frozen D6 blastocyst transfer, tubal infertility factor, presence of endometriosis, good-quality embryo transfer, the patient’s ovarian reserve (AMH level in ng/mL), IVF/ICSI rank, and the endometrium thickness (Table 3). Frozen D6 blastocyst transfer was independently associated with a significant increase in the LBR compared with fresh D6 transfer (OR = 2.54; 95% CI: [1.05–6.17]; *p* = 0.038), as well as a good-quality D6 blastocyst transfer, which had a significant positive impact on the LBR (OR = 3.85; 95% CI: [2.00–7.45]; *p* < 0.001*).* Conversely, advanced women’s age at the time of the oocyte retrieval was significantly associated with reduced chances of live birth (OR = 0.91; 95% CI: [0.86–0.96]; *p* = 0.002).

**Table 3:** Prognosis factors of live birth

	Multiple logistic regression analysis		
Parameters*	**Odds ratio**	**95% CI**	***p*** **-value**
Age at retrieval (y.o.)	0.91	0.86–0.96	0.002
Frozen versus fresh ET	2.54	1.05–6.17	0.038
Tubal factor	0.92	0.50–1.68	0.773
Endometriosis factor	0.94	0.59–1.48	0.773
Top- or good-quality embryo transfer^a^	3.85	2.00–7.45	< 0.001
AMH level ng/mL	0.974	0.87–1.09	0.974
IVF/ICSI rank	0.838	0.66–1.06	0.140
Endometrium thickness	0.930	0.82–1.05	0.242

y.o., years old; AMH, anti-Müllerian hormone; ET = Embryo transfer, IVF/ICSI, in vitro fertilization/intracytoplasmic sperm injection

* Variables included in the multiple logistic regression model

^a^ A top- and good-quality embryo were defined as (B3 AA, B4 AA, and B5 AA) and (B3 AB/BA/BB, B4 AB/BA/BB, and B5 AB/BA/BB), respectively

### Live birth occurrence according to D6 blastocyst morphology

For patients with live births (*n* = 104) after cycles of fresh or frozen D6 blastocyst transfer, a larger proportion of D6 transferred blastocysts were at expanded (E) stage 4 (49%), ICM grade B (81.7%), or TE grade B (71.2%) (Table 4). Interestingly, both the TE and ICM grades were significantly associated with live birth occurrence after both fresh and frozen D6 blastocyst transfers (*p* < 0.001 and *p* = 0.037, respectively).

**Table 4:** Morphology characteristics of transferred blastocysts by live birth status

Characteristic	No live birth (*n* = 792)	Live birth (*n* = 104)	*p-value*
**EH stage**			0.337
3	181 (22.9)	16 (15.4)	
4	362 (45.7)	51 (49.0)	
5	247 (31.2)	37 (35.6)	
6	2 (0.2)	0 (0.0)	
**ICM grade**			0.037
A	58 (7.3)^a, b^	13 (12.5)^a, b^	
B	636 (80.3) ^a, c^	85 (81.7) ^a, c^	
C	98 (12.4) ^b, c^	6 (5.8) ^b, c^	
**TE grade**			< 0.001
A	52 (6.5)^a, b^	23 (22.1) ^a, b^	
B	578 (73.0) ^a, c^	74 (71.2) ^a, c^	
C	162 (20.5)^b, c^	7 (6.7)^b, c^	

Values are n and %, EH = expansion and hatching; ICM = inner cell Mass; TE = trophectoderm

^Chi^ = Pearson’s chi-square test. Differences were considered statistically significant when the p-value was < 0.01 ^a^ between TE grade A versus TE grade B. ^b^ between TE grade A and TE grade C. ^c^ between TE grade B and TE grade C

Frozen overall D6 group = 1st warming cycle of a D6 blastocyst with possible previous fresh or frozen D5 embryo transfer. Elective frozen D6 = a subgroup among the Frozen overall D6 group that consisted of a 1st warming cycle of a D6 blastocyst, during a freeze-all cycle without any previous fresh or frozen D5 embryo transfers

## Discussion

Comparing to the few studies that have specifically addressed the best way to process D6 blastocyst transfers in both fresh and frozen cycles, our study would seem to be more original due to a higher number of confounding factors included in the analysis.

We here report a significantly higher LBR rate after frozen D6 blastocyst transfer (12.5%) compared with the LBR with fresh D6 transfer (5.5%), based on analysis of 896 single D6 blastocyst transfers. Moreover, after adjusting for potential confounders using multiple logistic regression analysis, frozen D6 blastocyst transfer remained independently associated with an increased LBR compared with that of fresh D6 transfer (OR 2.54 95% CI 1.05–6.17; *p* = 0.038). In terms of embryo morphology, the TE, and to a lesser extent, the ICM grade, were identified as the main morphological parameters predictive of live birth occurrence.

Our findings support the hypothesis previously raised in the literature suggesting that COS results in advanced endometrial development with an earlier window of implantation, which is not optimal for slowly developing D6 blastocysts in fresh transfers [15]. More specifically, COS induces (i) an advanced endometrial histology and (ii) premature down-regulation of the progesterone receptor, which leads to an advanced receptive phase. The degree of histologic advancement correlates with premature progesterone elevation and with implantation failure through an effect of embryo–endometrium asynchrony in fresh transfers. Shapiro and colleagues reported a significantly greater ongoing pregnancy rate in the frozen-thawed group than in the fresh group after the transfer of D6 blastocysts [15]. This observation is in line with the higher implantation and ongoing pregnancy rates following D6 blastocyst transfers in frozen cycles compared with those in fresh cycles [16]. The latter analysis reinforces our findings that deferring all D6 blastocyst transfers to a frozen cycle is the best way to increase the chances of LBR for these embryos due to improved endometrial receptivity.

However, several studies have not reported any significant differences in IVF outcomes between fresh versus frozen D6 blastocyst transfers. Yamamoto and colleagues found a significantly lower implantation rate in case of fresh versus frozen-thawed expanded D6 blastocyst transfers, but similar ongoing pregnancy rates per transfer [17]. Moreover, in the same study and contrary to our results, the authors found no difference concerning the implantation rate when comparing the first fresh D6 transfer with the first frozen D6 transfer [15.7% (24/153) vs. 28.6% (8/28), respectively; *p* = 0.17]. We hypothesize that the same comparison on larger series may have led to a significant difference between these two groups. In their recent study considering LBRs as the primary outcome and including 80 fresh D6 expanded blastocyst (≥ B4) transfers versus 180 frozen-thawed D6 transfers, Loubersac’s team found no significant difference in the LBR between the two groups (16.3% vs. 17.2%, respectively). However, in this study, some couples (77 and 153 in the fresh and frozen D6 groups, respectively) underwent several transfers (80 and 180 D6, respectively) [7]. As similar pregnancies outcome are reported after fresh or frozen D5 blastocysts transfers [2], the difference observed seems mainly related to an asynchrony between the embryo expanded at D6 and the endometrium rather than to the hormonal environment linked to the stimulation.

Thus, according to the data in the literature, the D6 blastocyst transfer strategy remains controversial. There have only been four published studies to date on this topic [7, 16–18]. The lack of significance may be due to small sample sizes, various statistical analyses with confounding factors (age of the patient, embryo quality, etc.), and heterogeneous main outcomes considered by the authors (usually the clinical pregnancy rate and not the live birth rate).

Low pregnancy rates related to both fresh and frozen D6 blastocysts transfer compared to D5 embryos reflect poor intrinsic embryo quality, i.e., they are less competent at implantation. These data corroborate the previous suggestion [18] that the delay in reaching blastocyst expansion in D6 could be followed by delayed hatching that is not synchronized with the endometrium during fresh as well as perhaps also in frozen cycles. In case of frozen D6 blastocyst cycle, tailoring endometrial preparation optimally can lead to higher live birth rates. Further large and prospective trial are needed to settle which progesterone regimen lead to significant improvement in IVF outcomes for day 6 embryos. Regarding the impact of embryo quality at D6, selecting the best morphological quality blastocyst for the first transfer in frozen cycles can lead to an increase in the implantation rates resulting in LBR improvement. More specifically, we found that both TE and ICM grades are the most important morphological parameters significantly associated with LBR after D6 blastocyst transfer. Our findings fuel the controversy concerning the best predictive factor of live birth at the blastocyst stage among blastocoel expansion, ICM and TE quality, either in fresh [19, 20] or in frozen transfer cycles [21], which are usually considered in D5 blastocysts. Van den Abbeel’s team has reported that blastocoel expansion should be considered first among the three morphological parameters when selecting a blastocyst for transfer, as they found that it was the parameter with the highest predictive value of live birth after transfer of a blastocyst expanded on D5 [22]. Our study, considering only D6 blastocysts, has shown that the TE grade has a greater predictive value than the ICM for selecting the best blastocyst before embryo replacement.

Chromosomal abnormalities are known to be highly correlated with implantation failure and early miscarriage. Interestingly, comparison of frozen euploid D5 versus D6 blastocyst transfer outcomes highlights that the clinical pregnancy rate remains significantly higher after D5 blastocyst than after D6 embryo transfers [23]. On the other hand, for some authors, the implantation potential of euploid embryos was the same, despite different morphologies and independent of the day of expansion (days 5, 6, or 7) [24]. In this controversial context, the ploidy status of D6 embryos does not appear to be the sole explanation for their lower pregnancy potential compared with D5 embryos. In our study, the ploidy status of the D6 blastocysts was not evaluated due to the non-authorization in France to carry out a PGT-A on embryos before transfer. Nevertheless, the complex pathophysiological mechanisms leading to such slow-growing D6 blastocysts remain to be elucidated. For this purpose, further studies are needed to explore the molecular events involved in embryo development and implantation. In the past two decades, advances in biotechnology have led to the emergence of new high-throughput techniques, grouped under the term ‘omics’ approaches (genomics, transcriptomics, proteomics, and metabolomics). The simultaneous analysis of multiple molecular entities provided by omics is crucial for the development of tools for a deeper understanding of embryo development and implantation as well as pathological situations, with the aim of improving patient issues during their ART management.

Some authors have proposed that embryos with developmental delays at D5 (morulae or early cavitating blastocyst) should no longer be cultured until D6 but rather that they should be transferred at D5. Indeed, they reported similar clinical outcomes using this strategy compared with those obtained after frozen D6 expanded blastocyst transfers [25, 26]. However, the heterogeneity of these results due to the diversity of the evaluated parameters in terms of clinical outcomes (cPR, LBR) does not allow a conclusion to be drawn concerning this embryo transfer strategy. When only non-expanded blastocysts (grades B1 or B2) are available at D5, a morphological assessment at D6 can improve embryo selection to increase positive clinical issues after embryo transfer.

Our experience, after 896 D6 blastocyst transfers, allowed us to change our embryo transfer policy concerning the management of slow-growing blastocysts, postponing every fresh D6 transfer to a frozen cycle. Our study adjusted for multiple confounders and implemented a subgroup analysis of the first FET of D6 blastocysts. We cannot rule out that the subgroup of fresh D6 transfer was related to women with significantly poorer ovarian reserves than those in the frozen overall group. Nonetheless, the LBR remained statistically higher in the Elective frozen D6 group compared to the LBR of the fresh D6 group following the 1st embryo transfer of a slow-growing D6 blastocyst. Despite a robust statistical analysis, our study suffers from its retrospective design. While the number of fresh cycles can be considered to be quite small, the consistency of our results was ensured by the homogeneity of the two subgroups. A randomized clinical trial comparing fresh versus frozen D6 transfers could be the best study design to further confirm these findings.

## Conclusion

To the best of our knowledge, this study is the first well-conducted approach to compare live birth rates between fresh versus frozen-thawed D6 embryo transfers considering a complete D6 blastocyst morphological characterization and an adjustment based on confounding factors. Regarding the literature data, it is preferable to transfer D5 blastocysts rather than D6 blastocysts during both fresh and frozen cycles, irrespective of the embryo morphology. Despite their lower potential, D6 blastocysts are usually used for transfer, especially in cases where only these types of embryos are obtained after prolonged culture. However, our results support the need to modify transfer policies, with cryopreservation of all D6 blastocysts instead of being transferred in fresh cycles. With this strategy, the live birth rates of first embryo transfers may be increased by allowing the best-quality blastocysts to be transferred in frozen cycles. Nevertheless, only randomized controlled trials considering larger series can confirm the benefit of transferring D6 blastocysts in frozen cycles.

## Data Availability

No datasets were generated or analysed during the current study.

## Abbreviations

AMH Anti: Müllerian Hormone
ART: Assisted Reproductive Technologies
HCG: Human Chorionic Gonatrophin
ICSI: Intra-cytoplasmic Sperm Injection
ICM: Inner Cell Mass
IVF: In Vitro Fertilization
PGT-A: Preimplantation Genetic Testing for Aneuploidy
TE: Trophectoderm
